# Supplementary material for: SAXS measurements of azobenzene lipid vesicles reveal buffer-dependent photoswitching and quantitative Z→E isomerisation by X-rays
Source: Nanophotonics. 2022 Apr 15;11(10):2361–8. doi: 10.1515/nanoph-2022-0053 (PMC11636378; doi:10.1515/nanoph-2022-0053)
Supplement: Supplementary file 1 — Supplementary Material [file j_nanoph-2022-0053_suppl.docx]

Supporting Information

Martina F. Ober^1^, Adrian Müller-Deku^2^, Anna Baptist^1^, Benjamin Ajanović^1^, Heinz Amenitsch^3^, Oliver Thorn-Seshold^2^, and Bert Nickel^1^

^1^ Faculty of Physics and CeNS, Ludwig-Maximilians-University München, Geschwister-Scholl-Platz 1, 80539 Munich, Germany

^2^ Department of Pharmacy, Ludwig-Maximilians-Universität München, Butenandtstraße 5-13, 81377 Munich, Germany

^3^ Institute of Inorganic Chemistry, Graz University of Technology, Stremayrgasse 9, 8010 Graz, Austria

Corresponding author: [*nickel@lmu.de](mailto:*nickel@lmu.de)

Content

[Notes: 1](#_Toc99463963)

[S1: Determination of the *E/Z* ratio of azo-PC by UV-Vis and HPLC 2](#_Toc99463964)

[S2: Custom-built UV-A/blue light pump – x-ray probe setup 6](#_Toc99463965)

[S3: cis-enriched azo-PC SUVs do not spontaneously isomerize to trans 7](#_Toc99463966)

[S4: Photoswitching azo-PC SUVs in PBS and in TE buffer via UV and blue light 8](#_Toc99463967)

[S5: Comparison of the Z>E switching of azo-PC SUVs in deionized water
 via blue light and from x-rays 9](#_Toc99463968)

[S6: Small angle X-ray scattering (SAXS) analysis 11](#_Toc99463969)

[S7: Higher x-ray energies for SAXS 14](#_Toc99463970)

[S8: Head-to-head distance of azo-PC membranes depends on the
 percentage of azo-PCs in *cis*-state 16](#_Toc99463971)

[S9: Photoswitching azo-PC SUVs in NaCl solution via UV and blue light 17](#_Toc99463972)

# **Notes:**

(1) ***trans*- (or *E-*) and *cis* (or *Z-*)** are the two isomer states of any single azo-PC molecule.

(2) On a **population** level, samples of azo-PC are denoted as e.g. "all-*trans*" or "80% *cis* " to indicate isomer ratios in the sample.

(3) **Photostationary states ("PSSs")** are equilibrium *E/Z* ratios reached under saturating illumination at given wavelengths, which depend on factors including the absorption coefficients of the *E & Z* isomers at that wavelength in their molecular environment, and the environment-dependent quantum yields of *E>Z* and *Z>E* photoisomerisation at that wavelength.

Therefore, the PSS at a given wavelength can be different depending on whether the photoswitch is in a molecular solution in a certain solvent (e.g. in a dilute solution in chloroform, typically < 100 µM concentration), or in a different solvent (e.g. same dilution but in methanol), or is present as a photolipid membrane.

Where PSSs are mentioned in the text, these are understood to be the PSSs established in SUVs, unless otherwise specified.

# **S1: Determination of the *E/Z* ratio of azo-PC by UV-Vis and HPLC**

Figure S1 – UV-Vis spectroscopy (all as diluted molecular solutions, all in >99% methanol). **a.** Overlay of the UV-Vis spectra of azo-PC and of model photoswitch FAzoM, showing their equivalence when in molecular solution. **b.** UV-Vis spectra of FAzoM samples with different E/Z ratios, given as %Z (FAzoM at 19 µM). **c.** Calibration curve of absorbance at 335 nm as a function of %Z (FAzoM at 19 µM). **d.** UV-Vis measurements of azo-PC before and after 365 nm illumination (stock in chloroform was illuminated, and aliquots were diluted into methanol for UV-Vis measurement).

Our first aim was to establish a calibration curve relating photolipid membrane ("p-membrane") isomer ratio to the thickness determined by SAXS, so we could then use SAXS thickness measurements to determine the p-membrane isomer ratio in test samples during photoswitching and during X-ray administration.

We did not succeed in reproducibly calibrating and measuring *trans:cis* ratios for intact p-membranes by H-NMR, by HPLC, or by UV-Vis. Therefore, we required another method to determine p-membrane isomer composition for generating the SAXS calibration curve.

**Procedure**

For calibrations, we prepared SUVs from samples of azo-PC with predefined *trans:cis* ratios, which were established by mixing two chloroform solution stocks each of known isomer composition (all*-trans* and mostly*-cis*) in varying proportions*.* The all-*trans* stock is accessible by thermal relaxation of a solution of azo-PC (e.g. 80°C overnight in a closed vessel). A mostly-*cis* stock was accessed by saturating UVA illumination of a solution of azo-PC in chloroform. The remaining task was then to measure the %Z content in the mostly-*cis* chloroform stock, in order to know the *E/Z* ratios in the SUVs.

High-performance liquid chromatography (HPLC) of azo-PC stocks, aiming to separate *E* and *Z* isomers and quantify their relative proportions by inline detection (eg. diode array detector), also proved poorly reproducible, due to the zwitterionic surfactant nature of the compound.

However, the simpler, non-charged and non-polar compound FAzoM (Scheme S1) that contains the same chromophore as azo-PC, can be analysed quantitatively and reproducibly for *cis:trans* ratio by HPLC. Therefore, we established a calibration series of UV-Vis spectra for different *cis:trans* ratios of FAzoM that were checked by HPLC (Fig S1b-c); confirmed that spectra for FAzoM and AzoPC were essentially identical so spectra of samples of AzoPC could be mapped onto those of samples of FAzoM to determine isomer ratios (Fig S1a); and finally derived the percentage of *cis*-AzoPC in the mostly-*cis* chloroform stock by taking a UV-Vis spectrum of an aliquot of this stock diluted into methanol, and comparing it to the calibration series.

The synthesis and characterisation of the useful model compound FAzoM has, to our knowledge, not been reported before, so is provided below.

Scheme S1. FAzoM synthesis from FAzo and comparison to azo-PC structure

***FAzoM (methyl (E)-4-(4-((4-butylphenyl)diazenyl)phenyl) butanoate)***

A round bottom flask was charged with (*E*)-4-(4-((4-butylphenyl)diazenyl)phenyl) butanoic acid (FAzo [1]) (1 eq., 200 mg, 0.616 mmol) and 4 mL methanol was added. Acetyl chloride (1 eq., 44 μL, 48.4 mg, 0.616 mmol) was added dropwise. The flask was closed and stirred at 25 °C for 16 h. The methanol was removed by evaporation and the crude product was purified by column chromatography using 9:1 hexanes:ethyl acetate eluent. The desired product FAzoM was obtained as an orange solid (187 mg, 0.553 mmol, 90%).

**^1^H NMR** (400 MHz, Chloroform-*d*) δ (ppm) = 7.84 – 7.82 (m, 4H), 7.31 (d, *J* = 8.1 Hz, 4H), 3.68 (s, 3H), 2.73 (t, *J* = 7.6 Hz, 2H), 2.69 (t, *J* = 7.6 Hz, 2H), 2.36 (t, *J* = 7.4 Hz, 2H), 2.01 (p, *J* = 7.5 Hz, 2H), 1.57 (p, *J* = 7.5 Hz, 2H), 1.39 (h, *J* = 7.4 Hz, 2H), 0.95 (t, *J* = 7.3 Hz, 3H). **^13^C NMR** (101 MHz, CDCl_3_) δ (ppm) = 173.9, 151.4, 151.1, 146.5, 144.6, 129.3, 129.2, 123.0, 122.9, 51.7, 35.7, 35.1, 33.6, 33.5, 26.4, 22.5, 14.1. **LCMS(+):** t_ret_ = 8.0 min, [MH]^+^ = 339 Th. **HRMS (EI):** calc. for C_21_H_26_O_2_N_2_^+^ [M]^+^: 338.1994 Th; found: 338.1995 Th.

Next, it was first verified that the UV/Vis-spectra of azo-PC and FAzoM in molecular solution match well to each other (Fig S1a). After that, a 3.8 mM stock of FAzoM in chloroform was prepared; and 50 µL were transferred to several clear glass tubes. The FAzoM aliquots were irradiated for different durations with a 370 nm LED (3W H2A1-model from Roithner Lasertechnik; FWHM bandwidth ca. 20 nm) to give a series of different *E/Z* ratios (longer illumination approaches more closely the PSS at 370 nm). The FAzoM *E/Z* ratios in each tube were determined by HPLC analysis (see **Methods** below). Subsequently, the FAzoM samples were diluted with methanol to a concentration of 19 µM (i.e. >99% methanol) and UV-Vis spectra in molecular solution were measured. The spectra were normalized to the absorbance at the isosbestic point in methanol at 285 nm (Fig S1b) to correct for variations due to pipetting volumes (typically <5% correction), and the absorbance at 335 nm (maximum absorbance of the *E* isomer) was correlated to the HPLC-determined %*Z* in the sample, resulting in a linear correlation (Fig S1c):

A_335_ = -0.007923 × (%Z) + 0.7818 (R^2^ = 0.998)

Applying this calibration curve to the correspondingly diluted and normalised UV-Vis spectrum of the UV-illuminated stock of azo-PC in chloroform that we employed, we calculated its %*Z* to be 83% (Fig S1d). The thermally relaxed stock of azo-PC intended to be all-*trans* was also measured spectroscopically and confirmed to have %*Z* < 0.5%. Finally, the all-*trans* and the mostly-*cis* stocks of azo-PC were mixed in a range of proportions, before SUVs were prepared and membrane thicknesses measured by SAXS, as detailed in the following sections.

**Methods: HPLC** was performed on an Agilent 1100 SL coupled HPLC system with (a) a binary pump to deliver H_2_O:MeCN eluent mixtures containing 0.1% formic acid at a 1 mL/min flow rate, (b) Agilent 10 Prep-C18 Scalar 250 x 4.6 mm maintained at 30°C, whereby the solvent front eluted at t_ret_ = 2.3 min, and (c) an Agilent 1100 series diode array detector (DAD). For isomer separation, a gradient from 90% to 100% MeCN over 10 min, and holding at 100% MeCN until completion, was used. Under these conditions the *Z* isomer eluted at 5.2 min and the *E*-isomer at 10.3 min. For ratio determination, the signals in the 391 nm DAD trace were integrated, since this is an isosbestic point for FAzoM in >90% MeCN. **UV-Vis** absorption spectra in cuvette were acquired on a Varian CaryScan 60 (1 cm pathlength) using Hellma precison SUPRASIL quartz cells.

# **S2: Custom-built UV-A/blue light pump – x-ray probe setup**


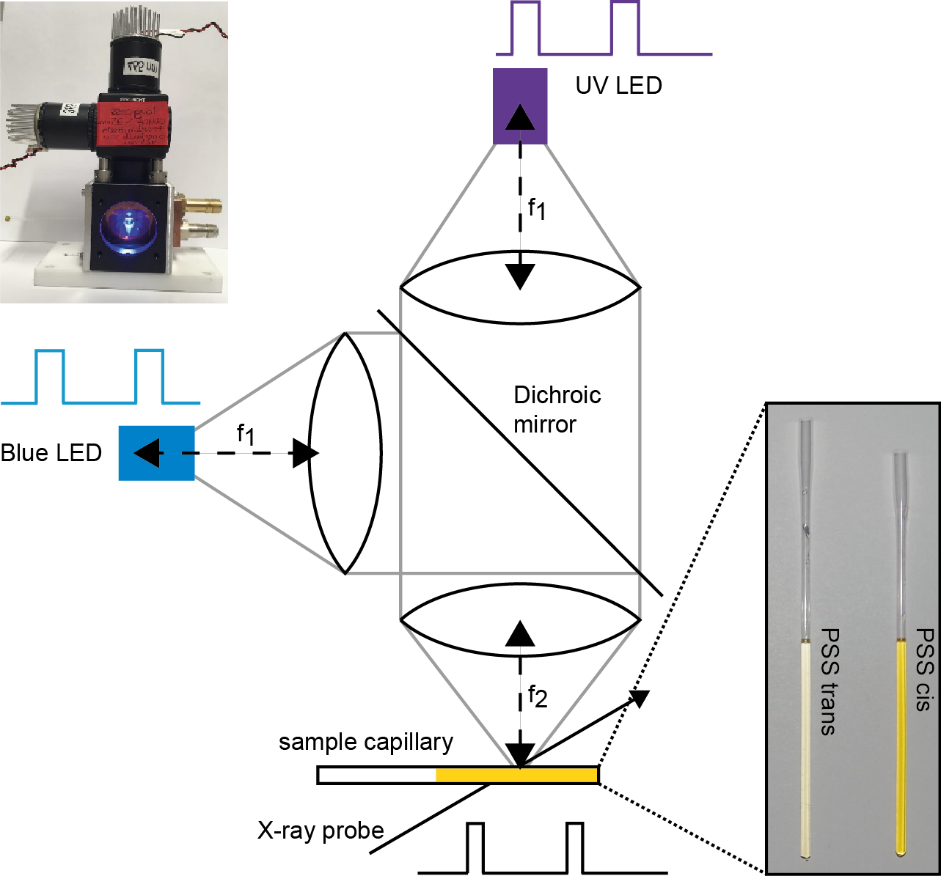


Figure S2: Schematic of our custom-built UV-A/blue light illumination setup used for illuminations during SAXS measurements. A photograph of the setup, and of two capillaries with azo-PC SUVs in the predominantly trans PSS at 465 nm (left) and predominantly cis PSS at 370 nm (right), are shown.

# **S3: cis-enriched azo-PC SUVs do not spontaneously isomerize to trans**


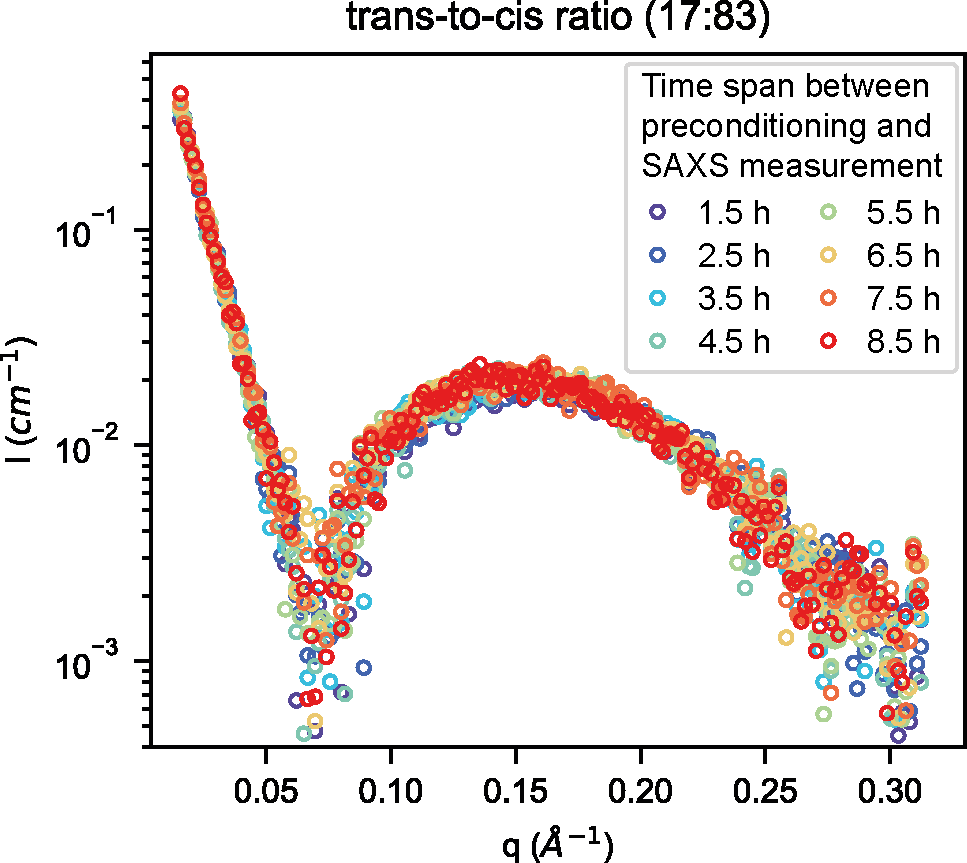


Figure S3: SAXS intensities for unilamellar azo-PC vesicles prepared with a predefined trans-to-cis ratio of (17:83) integrated over 1h with a time-lap between preconditioning and SAXS measurement as indicated in the legend.

Eight consecutive SAXS measurements of preconditioned trans-to-cis ratio of (17:83) azo-PC SUVs yield an identical SAXS signal. There is no sign of a spontaneous back reaction, i.e. cis-to-trans isomerization, over 8.5 h of monitoring after the preconditioning of the azo-PC SUVs was performed (we estimate that at least a 4% relaxation would be visible, so, we can offer an upper bound of 4% spontaneous relaxation over 8 hours, which translates to insignificant change during the active experiments). This finding is in agreement with the slow thermal relaxation rate for *Z-*para,para'-bis(alkyl)azobenzenes (days to weeks under physiological conditions) [2].

# **S4: Photoswitching azo-PC SUVs in PBS and in TE buffer via UV and blue light**


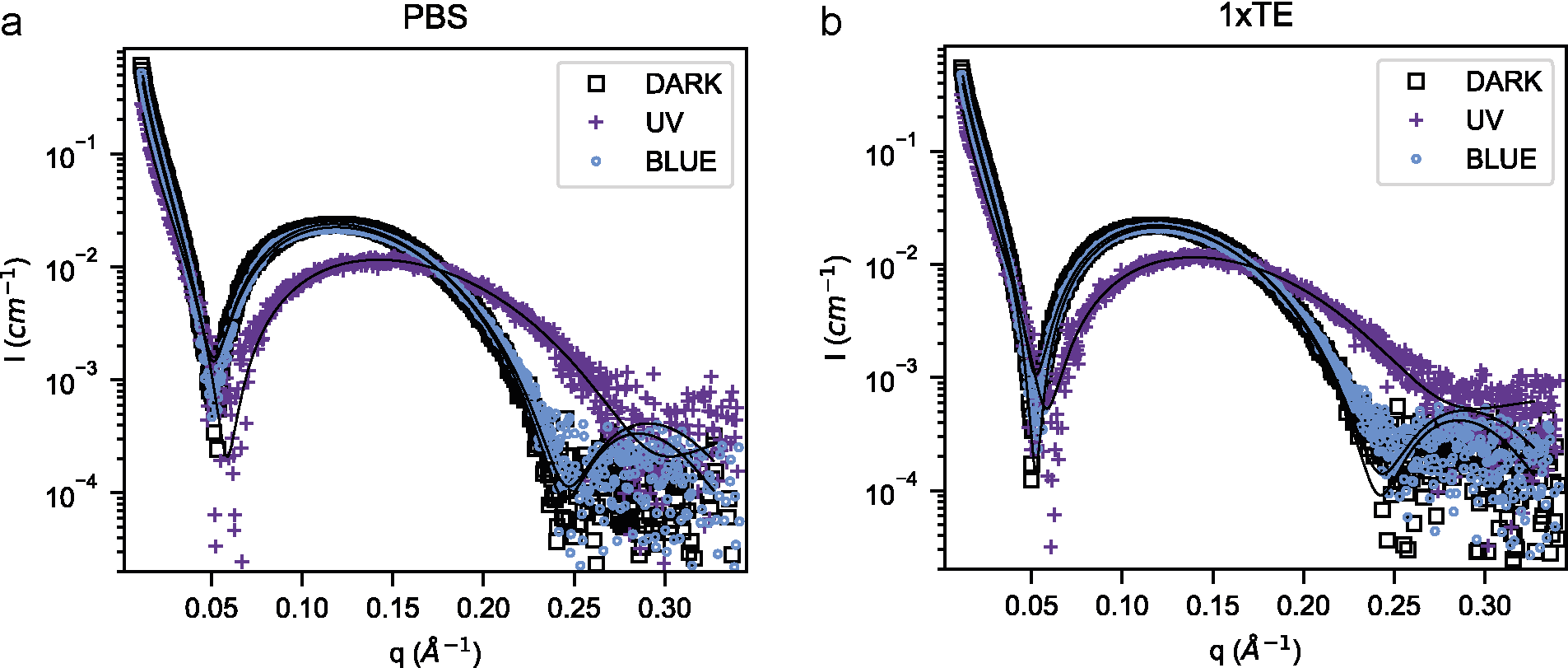


Figure S4: SAXS intensities for unilamellar azo-PC vesicles prepared in PBS buffer (a) and 1×TE buffer (b), for the dark-adapted state, and for photostationary states induced by extended UV-A or blue light illumination (each >300s) are shown as squares, crosses and circles respectively. Intensities are on an absolute scale. Note the crossing point at ca. q = 0.18, as expected for measurements with linear response in the absence of hysteresis, therefore indicating the reproducibility of these measurements.

**S5: Comparison of the Z>E switching of azo-PC SUVs in deionized water from blue light (partial Z>E switching) and from x-rays (full Z>E switching)**


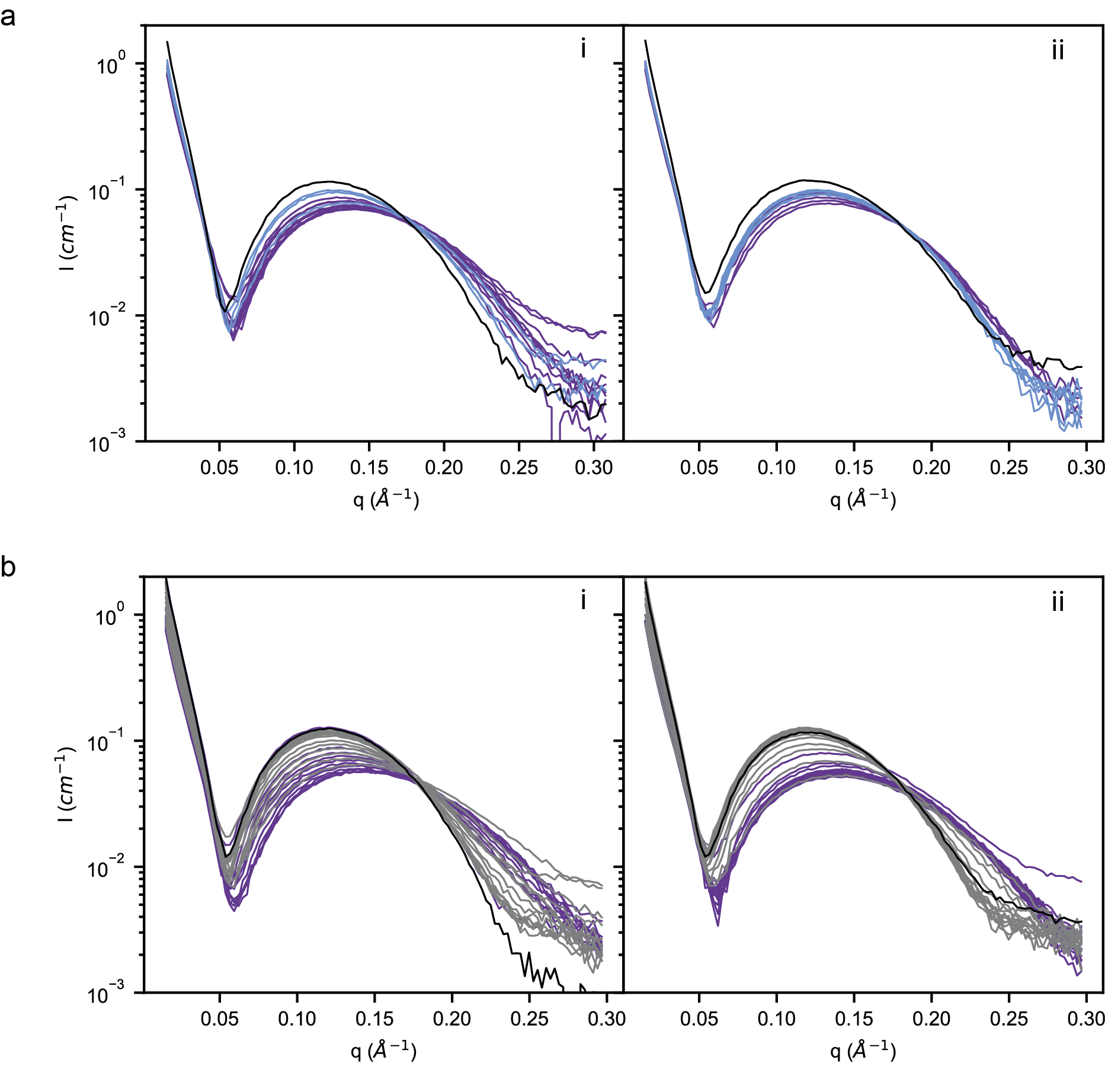


Figure S5: **Switching of photolipid membranes in DI water via blue light and via x-rays** (data related to Figures 2-3). **a(i)** In-situ SAXS measurements on azo-PC SUVs initially in the dark state (all-trans, black), then during 120 s of UV illumination (purple, increased %Z), then during 30 s of blue illumination (blue, %Z intermediate between UV and dark conditions). **a(ii)** Similar in-situ SAXS measurements on azo-PC SUVs as in **a(i)**, but using 60 s of UV illumination and 60 s of blue illumination. **b(i)** In-situ SAXS measurements on azo-PC SUVs initially in the dark state (black, all-trans) then during 105 s of UV illumination (purple) then during subsequent x-ray exposures (grey). **b(ii)** Similar in-situ SAXS measurements on azo-PC SUVs as in **b(i)**, but using 205 s of UV illumination. Note again the crossing points at ca. q = 0.18, indicating the reproducibility of these measurements (cf Fig S3). X-ray exposure and delay times are given in Table S1.

|  | Number of x-ray exposures | x-ray exposure time [s] | x-ray off time [s] | $t_{UV}[s]$ | $t_{blue}[s]$ |
| --- | --- | --- | --- | --- | --- |
| Fig S4a(i) | 15 | 5 | 5 | 120 | 30 |
| Fig S4a(ii) | 12 | 5 | 5 | 60 | 60 |
| Fig S4b(i) | 40 | 5 | 5 | 205 |  |
| Fig S4b(ii) | 30 | 5 | 5 | 105 |  |
| Fig. 3a (8keV) | 20 | 1 | 19 | 160 |  |

Table S1 Detailed timing parameters for SAXS experiments for data in Figures.

# **S6: Small angle X-ray scattering (SAXS) analysis**

All bilayer parameters are obtained from model fits of the total scattering intensity *I(q*) to an electron density profile $\Delta\rho\left( z \right)$ composed of three Gaussians.

$$\Delta\rho\left( z \right)=\Delta\rho_{H}\exp\left[ -\frac{\left( z-z_{H} \right)^{2}}{2\sigma_{H}^{2}} \right]+ {\Delta\rho}_{CH}\exp\left[ -\frac{\left( z \right)^{2}}{2\sigma_{CH}^{2}} \right]+ {\Delta\rho}_{H}\exp\left[ -\frac{\left( z+z_{H} \right)^{2}}{2\sigma_{H}^{2}} \right]$$

Here, Δ$\rho_{H}$ is the scattering length contrast of the lipid head groups compared to water, i.e. $\Delta\rho\left( z \right)=\Delta\rho_{H}- \Delta\rho_{w}$. $z_{H}$ is the spatial peak offset of the head centers in respect to the center of the bilayer, and $\sigma_{H}$ is the corresponding variance of the Gaussian functions. $\Delta\rho_{CH}$ is the scattering length contrast of the lipid chains and $\sigma_{CH}$ the variance of the Gaussian function describing the chain region. For the data shown in Figure 1b and Figure S4 we used a power law to account for additional background. Model fitting was achieved by running the software-internal population-based DREAM algorithm using the software package SasView (<http://www.sasview.org/>). The head-to-head distance uncertainty composes of purely statistical parameter errors corresponding to the 95% (two-$\sigma$) confidence interval and uncertainties reflecting the robustness of $d_{HH}$ with respect to the fitting model, which is approximately $\pm0.5 Å .$


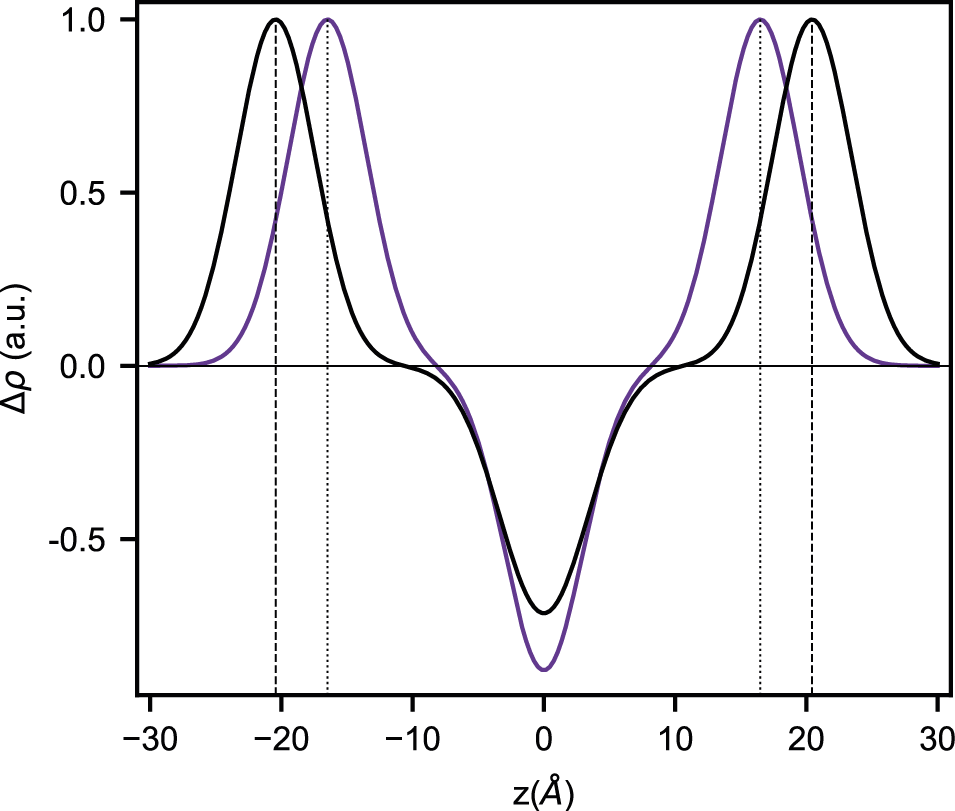


Figure S6 Exemplary electron density profiles Δρ(z) corresponding to the 0% cis (all-trans, dark state, black) and 80% cis (mostly-cis, UV-illuminated, purple) p-membranes (SAXS data in Figure 1b) as a function of distance z from the bilayer center. The bilayer thicknesses of the all-trans-azo-PC membrane (dashed lines) and of the 80% cis-azo-PC membrane (dotted lines) are highlighted.

Detailed fit parameters: Δ$\rho_{H}=1$ (Fixed), $\sigma_{H}=3Å$ (Fixed)

| Data | $z_{H}$ [$Å$] | Δ$\rho_{CH}$[a.u.] | $\sigma_{CH}[Å]$ | ${\chi^{2}}_{red}$ |
| --- | --- | --- | --- | --- |
| Fig. 1b 0$\pm5$% cis | 20.44$\pm$0.04 | -0.71$\pm$0.00 | 3.37$\pm$0.02 | 1.96 |
| Fig. 1b 10$\pm5$% cis | 20.75$\pm$0.03 | -1.08$\pm$0.00 | 2.25$\pm$0.01 | 2.05 |
| Fig. 1b 19$\pm5$% cis | 20.21$\pm$0.03 | -0.87$\pm$0.00 | 2.97$\pm$0.01 | 3.48 |
| Fig. 1b 39$\pm5$% cis | 19.16$\pm$0.03 | -0.87$\pm$0.00 | 3.00$\pm$0.01 | 3.49 |
| Fig. 1b 58$\pm5$% cis | 17.34$\pm$0.04 | -0.86$\pm$0.00 | 3.00$\pm$0.01 | 1.87 |
| Fig. 1b 83$\pm5$% cis | 16.49$\pm$0.04 | -0.88$\pm$0.00 | 3.00$\pm$0.01 | 1.30 |
| Fig. 3a top I | 17.91$\pm$0.09 | -2.58$\pm$0.05 | 1.08$\pm$0.01 | 0.76 |
| Fig. 3a top II | 18.96$\pm$0.04 | -2.53$\pm$0.06 | 1.06$\pm$0.01 | 1.64 |
| Fig. 3a top III | 19.75$\pm$0.05 | -4.56$\pm$0.10 | 0.57$\pm$0.01 | 2.00 |
| Fig. 3a top IV | 20.26$\pm$0.06 | -3.07$\pm$0.03 | 0.83$\pm$0.01 | 2.76 |
| Fig. 3a top V | 20.77$\pm$0.05 | -1.77$\pm$0.02 | 1.39$\pm$0.01 | 3.46 |
| Fig. 3a top VI | 21.02$\pm$0.04 | -4.07$\pm$0.03 | 0.61$\pm$0.01 | 2.10 |
| Fig. 3a bottom I | 19.09$\pm$0.06 | -0.93$\pm$0.01 | 2.71$\pm$0.02 | 5.81 |
| Fig. 3a bottom II | 19.00$\pm$0.06 | -2.45$\pm$0.02 | 1.02$\pm$0.01 | 6.10 |
| Fig. 3a bottom III | 18.86$\pm$0.06 | -0.65$\pm$0.01 | 4.01$\pm$0.04 | 5.90 |
| Fig. 3a bottom IV | 19.10$\pm$0.05 | -0.58$\pm$0.01 | 4.34$\pm$0.04 | 5.19 |
| Fig. 3a bottom V | 19.16$\pm$0.06 | -6.05$\pm$0.02 | 0.41$\pm$0.01 | 5.02 |
| Figure S3a dark (PBS) | 21.48$\pm$0.03 | -1.20$\pm$0.00 | 2.39$\pm$0.01 | 0.32 |
| Figure S3a PSS_UV_ (PBS) | 17.33$\pm$0.03 | -1.01$\pm$0.00 | 3.14$\pm$0.01 | 3.89 |
| Figure S3a PSS_blue_ (PBS) | 21.11$\pm$0.01 | -0.95$\pm$0.01 | 3.13$\pm$0.00 | 6.11 |
| Figure S3b dark (1xTE) | 21.45$\pm$0.04 | -0.98$\pm$0.00 | 2.76$\pm$0.01 | 0.33 |
| Figure S3b PSS_UV_ (1xTE) | 17.71$\pm$0.02 | -0.98$\pm$0.00 | 3.14$\pm$0.00 | 8.73 |
| Figure S3b PSS_blue_ (1xTE) | 21.22$\pm$0.04 | -0.86$\pm$0.01 | 3.12$\pm$0.02 | 0.32 |
| Figure S4a (i) dark | 20.54$\pm$0.02 | -0.99$\pm$0.00 | 2.74$\pm$0.01 | 2.25 |
| Figure S4a (i) PSS_UV_ | 17.66$\pm$0.01 | -0.97$\pm$0.00 | 3.16$\pm$0.02 | 2.50 |
| Figure S4a (i) PSS_blue_ | 19.46$\pm$0.02 | -0.95$\pm$0.00 | 3.13$\pm$0.02 | 2.43 |
| Figure S4a (ii) dark | 20.81$\pm$0.07 | -0.99$\pm$0.01 | 2.57$\pm$0.02 | 0.12 |
| Figure S4a (ii) PSS_UV_ | 18.24$\pm$0.03 | -0.97$\pm$0.00 | 3.17$\pm$0.01 | 0.53 |
| Figure S4a (ii) PSS_blue_ | 19.52$\pm$0.02 | -0.95$\pm$0.00 | 3.10$\pm$0.01 | 0.98 |
| Figure S4b (i) dark | 21.40$\pm$0.02 | -0.99$\pm$0.01 | 2.42$\pm$0.01 | 1.95 |
| Figure S4b (i) PSS_UV_ | 17.09$\pm$0.01 | -0.99$\pm$0.00 | 3.17 $\pm$0.01 | 4.20 |
| Figure S4b (i) x-ray | 21.15$\pm$0.02 | -0.82$\pm$0.01 | 3.14 $\pm$0.01 | 5.78 |
| Figure S4b (ii) dark | 21.10$\pm$0.02 | -0.81$\pm$0.01 | 2.99$\pm$0.01 | 3.11 |
| Figure S4b (ii) PSS_UV_ | 17.40$\pm$0.02 | -0.82$\pm$0.01 | 3.75$\pm$0.02 | 4.95 |
| Figure S4b (ii) x-ray | 21.32$\pm$0.02 | -0.83$\pm$0.00 | 3.04$\pm$0.01 | 6.15 |
| Figure S7 dark | 20.61$\pm$0.1 | -0.85$\pm$0.01 | 3.00$\pm$0.02 | 1.61 |
| Figure S7 PSS_UV_ | 18.52$\pm$0.2 | -0.88$\pm$0.01 | 3.00$\pm$0.02 | 1.70 |
| Figure S7 PSS_blue_ | 21.78$\pm$0.2 | -0.82$\pm$0.01 | 3.00$\pm$0.03 | 2.28 |

Table S2: Parameters obtained from least-squares fitting of SAXS data of 50 nm extruded azo-PC vesicles to a symmetrical, flat bilayer model. Errors indicate the purely statistical parameter uncertainties corresponding to a two-sigma confidence interval.

# **S7: Higher x-ray energies for SAXS**


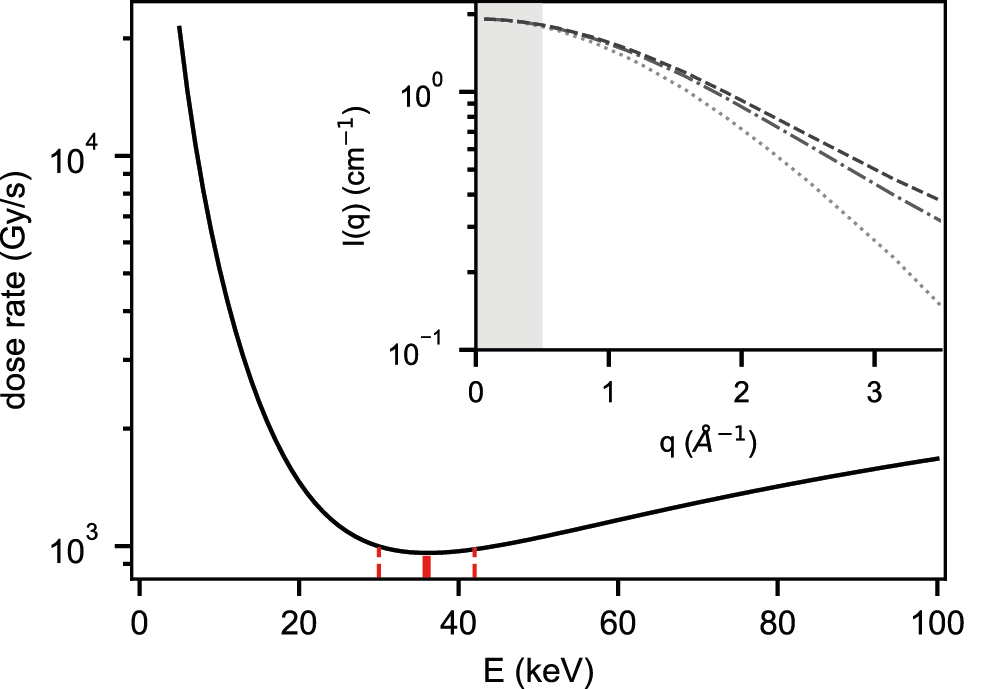


Figure S7 Energy dependent dose rate for water calculated for an optimal sample transmission of 37 % and a beam fluence of 10^12^ cts.s^-1^.mm^-2^. The dose rate minimum at 36 keV is highlighted in red. The inset shows the Rayleigh scattering intensity for carbon on absolute scale dependent on the scattering vector q for x-ray energies of 8 keV (dotted), 13 keV (solid), 17 keV (dashed-dotted), and 54 keV (dashed).

We stress the benefit of reducing the x-ray dose for solution-based BioSAXS experiments that using high-energy x-rays brings, as recently discussed for macromolecular crystallography by Dickerson and Garman [4]. The absorbed dose rate *D* can be calculated via the equation below [5]:

$D= \frac{E_{T}}{m}= \frac{I_{0}*E*t \left( 1-\exp\left( -\mu_{ic}\left( E \right)* \lambda\right) \right)}{V*\rho}= \frac{I_{0}*E*t \left( 1-\exp\left( -\mu_{ic}\left( E \right)* \lambda\right) \right)}{A* \lambda*\rho}$ (1)

Here, $E_{T}/m$ is the transferred energy per mass, $I_{0}$ the beam intensity, $E$ the x-ray energy, *t* the exposure time, $\mu_{ic}$the incoherent contribution of the attenuation coefficient [6], i.e. the Compton and the photoelectric contribution, $V$ the sample volume, $\rho$ its density and $\lambda$ its attenuation length; all these parameters are known and tabulated. Since the main volume fraction in SUV experiments is water, Figure S6 uses the energy dependence of the x-ray dose for water. As evident from Figure S6 the absorbed dose drastically drops for increasing energies in the regime $\boldsymbol{5}\boldsymbol{-}\boldsymbol{30} \boldsymbol{keV}$. After a minimum of $\boldsymbol{36} \boldsymbol{keV}$, the dose rate shows a mild increase for energies bigger than $\boldsymbol{40} \boldsymbol{keV}$. Such considerations clearly point to a favourable x-ray energy regime of $\boldsymbol{30}\boldsymbol{-}\boldsymbol{42} \boldsymbol{keV}$for SAXS experiments on radiation-sensitive samples. The dose rate is calculated using the mass attenuation coefficients from the NIST XCOM database [7].

Using higher x-ray energies does not imply per se a loss of scattering signal. In order to explain this, we plot the SAXS intensity for a carbon scattering centre in dependence of the scattering vector $q$ in $\left[ Å^{-1} \right]$ for three different x-ray beam energies$8.0 keV, 17.4 keV$, and$53.7 keV$ as dotted line, dashed-dotted line, dashed line, respectively, as inset in Figure S6 [8]. The intensity of a SAXS experiment on an absolute scale in$\left[ cm \right]^{-1}$ is given by the differential Rayleigh cross section$\frac{d\sigma_{R}}{d\Omega}$, i.e. the detection probability of an elastically scattered photon per unit solid angle and the number density $\rho$of the scatters:

$$I_{SAXS}\left( \theta\right)= \rho\frac{d\sigma_{R}}{d\Omega}$$

$$\frac{d\sigma_{R}}{d\Omega}= r_{e}^{2} P \left| f(\theta) \right|^{2}$$

Here, the Thomson radius is denoted as $r_{e}$, $f(\theta)$ is the atomic form factor, and $P$ the polarization of the x-ray beam. From the inset of Figure S6 it is readily apparent that there is almost no difference in the number of elastically scattered photons, within the SAXS regime ($0- 0.5 Å)$ for the x-ray energies discussed. The same is also true for the differential Compton scattering cross section within the SAXS regime (data not shown), i.e. the Compton scattering does not result in additional background signal. This is maybe surprising in view of the total Compton scattering cross section, which becomes larger than the photoelectric effect and the Rayleigh scattering beyond 28 keV. However, for SAXS we are only interested in the Compton forward scattering, which remains low. In agreement with our experiments shown in Figure 2a, high energy SAXS yields high quality data even for weakly scattering samples.

# **S8: Head-to-head distance of azo-PC membranes depends on the percentage of azo-PCs in *cis*-state**

We obtain a linear correlation of the head-to-head distance of azo-PC membranes with the percentage of azo-PC that is in the *cis* isomer state (see Fig. 1c),

$$d_{HH}= 42.7-0.1226 \times(\%cis)$$

by least-squares fitting of five different membrane compositions: 10%, 19%, 39%, 58% and 83% *cis* isomer. As the measured head-to-head distances $d_{HH}$for the dark-adapted state are not well-reproducible (Fig 1c, Fig 2b), the first data point (0% cis) was treated as an outlier and omitted during the linear fitting.

Applying this calibration curve to the measured head-to-head distances, we calculated *cis* isomer fractions as given in Table S3.

|  | PBS / 1xTE | | Deionized water | |
| --- | --- | --- | --- | --- |
|  | $d_{HH}(Å)$ | %cis isomer | $d_{HH}(Å)$ | %cis isomer |
| dark-adapted | $42.9\pm0.2$ | 0% ± 2% | $41.9\pm0.9$ | 7% ± 11% |
| ${PSS}_{UV}$ | $35.0 \pm0.4$ | 63% ± 5% | $34.8 \pm0.6$ | 64% ± 7% |
| ${PSS}_{blue}$ | $42.3 \pm0.4$ | 3% ± 5% | $39.0 \pm0.3$ | 30% ± 4% |
| ${PSS}_{x-ray}$ |  |  | $42.5\pm0.3$ | 2% ± 4% |

Table S3 Relevant mean head-to-head distances of azo-PC membranes obtained for various photostationary states (PSS) and their calculated percentages of cis isomer.

# **S9: Photoswitching azo-PC SUVs in NaCl solution via UV and blue light**


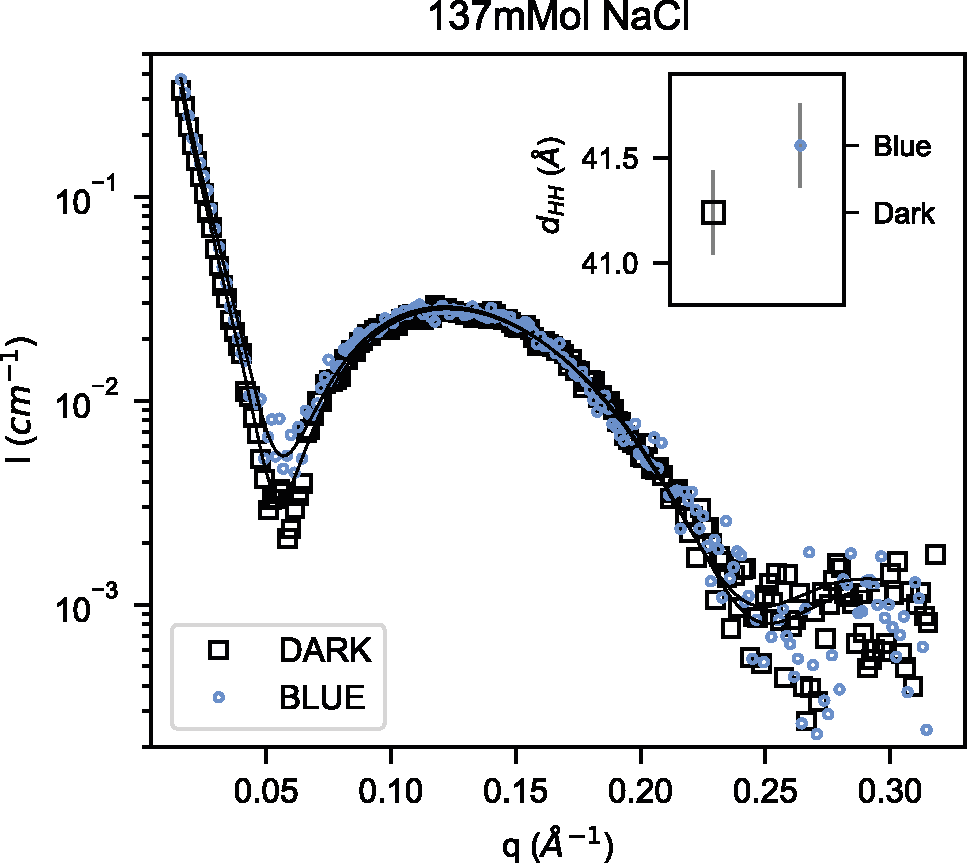


Figure S 8: SAXS intensities for unilamellar azo-PC vesicles prepared in 137 mM NaCl solution for the dark-adapted state, and the photostationary state induced by extended blue light illumination (>300s) are shown as squares and circles respectively. The Intensity of the blue photostationary state is vertically offset for clarity. Head-to-head distances (d_HH_) obtained for azo-PC SUVs in NaCl solution are shown in the inset. The dark-adapted state, and photostationary states induced via blue light are labelled accordingly.

References:

1. Frank, J. A.; Moroni, M.; Moshourab, R.; Sumser, M.; Lewin, G. R.; Trauner, D., Photoswitchable fatty acids enable optical control of TRPV1. Nature Communications 2015, 6 (1), 7118.

2. Morstein, J.; Trauner, D., Chapter Eleven - Photopharmacological control of lipid function. In *Methods in Enzymology*, Chenoweth, D. M., Ed. Academic Press: 2020; Vol. 638, pp 219-232.

3. Komorowski, K.; Salditt, A.; Xu, Y.; Yavuz, H.; Brennich, M.; Jahn, R.; Salditt, T., Vesicle Adhesion and Fusion Studied by Small-Angle X-Ray Scattering. Biophys J 2018, 114 (8), 1908-1920.

4. Dickerson, J. L.; Garman, E. F., The potential benefits of using higher X-ray energies for macromolecular crystallography. Journal of Synchrotron Radiation 2019, 26 (4), 922-930.

5. Attix, F. H., Introduction to Radiological Physics and Radiation Dosimetry. Wiley VCH: 2004.

6. Berger, M. J., Hubbell, J.H., Seltzer, S.M., Chang, J., Coursey, J.S., Sukumar, R., Zucker, D.S., and Olsen, K., XCOM: Photon Cross Section Database (version 1.5). National Institute of Standards and Technology, Gaithersburg, MD.: 2010.

7. Berger, M. J.; Hubbell, J. H.; Seltzer, S. M.; Chang, J.; Coursey, J. S.; Sukumar, R.; Zucker, D. S.; and Olsen, K., XCOM: Photon Cross Sections Database. National Institute of Standards and Technology, Gaithersburg, MD, USA 1998.

8. Hubbell, J. H.; Veigele, W. J.; Briggs, E. A.; Brown, R. T.; Cromer, D. T.; Howerton, R. J., Atomic form factors, incoherent scattering functions, and photon scattering cross sections. Journal of Physical and Chemical Reference Data 1975, 4 (3), 471-538.
